# NOD2/CARD 15 gene mutations in patients with Familial Mediterranean Fever

**DOI:** 10.1186/1546-0096-9-S1-P291

**Published:** 2011-09-14

**Authors:** Y Berkun, A Karban, S Padeh, Y Shinar, E Pras, M Lidar, A Livneh, Y Bujanover

**Affiliations:** 1 Department of Pediatrics A and Pediatric Rheumatology, Edmond & Lily Safra Children’s Hospital, Chaim Sheba Medical Center, Tel Hashomer, Sackler School of Medicine, Tel Aviv University, Tel Aviv, Israel

## Background

Familial Mediterranean fever (FMF) and Crohn’s disease (CD) are autoinflammatory disorders, associated with genes (MEFV and NOD2/CARD15), encoding for regulatory proteins, important in innate immunity, apoptosis, cytokine processing and inflammation. While mutations in the MEFV gene were shown to modify CD, the role of NOD2/CARD 15 gene mutations in the FMF disease phenotype was never studied before.

## Methods

The cohort consisted of 103 consecutive children with FMF, followed in a single referral center. NOD2/CARD15 genotypes were analyzed in all patients and 299 ethnically matched unaffected controls. Demographic data, clinical characteristics and disease course of FMF patients with and without NOD2/CARD 15 mutation were compared.

## Results

A single NOD2/CARD 15 mutation was detected in 10 (9.7%) FMF patients and 26 (8.7%) of controls. No homozygotes or compound heterozygotes were discovered in the 2 groups. FMF patients carrying a NOD2/CARD 15 mutation had higher rate of erysipelas-like erythema and acute scrotum attacks and a trend for higher rate of colchicine resistence and a more severe disease as compared to patients without mutations.

## Conclusion

NOD2/CARD 15 mutations are not associated with a susceptibility to develop FMF, yet the presence of these mutations in FMF patients appears to be associated with a trend to a more severe disease.

